# P04.20. Health informaton seeking, trust in information sources and use of complementary and alternative medicine

**DOI:** 10.1186/1472-6882-12-S1-P290

**Published:** 2012-06-12

**Authors:** L Rutten, S Stevenson

**Affiliations:** 1 SAIC, Inc, National Cancer Institute, Frederick, USA; 2 Nourish - Integrative Medicine Services, Cincinnati, USA

## Purpose

Patient engagement in health information seeking is associated with beneficial social, emotional, and health outcomes. We analyzed data from the 2008 Health Information National Trends Survey to characterize, and compare patterns of health information seeking and trust in sources of health information among persons who use complimentary and alternative (CAM) approaches to health and medicine and those who do not.

## Methods

SUDAAN was used to calculate population estimates. Weighted frequencies along with crosstabulation and chi-square analyses were conducted.

## Results

A significantly higher percentage of CAM users (83.2%) reported health information seeking, compared with non-users (65.4%). Differences in health information sources by CAM use were also observed. A higher percentage of CAM users (17.4%) reported use of print resources (e.g. books, newspapers) and the internet (63.5%), compared to non-users (15.9% and 60.6%, respectively). CAM users (11.6%) reported use of healthcare providers less frequently than non-users (15.0%). Reliance on friends and family for health information was similar among CAM users (3.9%) and non-users (4.2%). An interesting pattern of differences in trust in health information sources by CAM use was also observed. CAM users generally reported less trust in specified sources of information (e.g. family/friends, television, radio, newspaper, government). Additionally, a lower percentage of CAM users (60.4%) reported trust in healthcare professionals than non-users (71.2%). The only source of health information wherein greater trust was reported by CAM users (21.8%) compared with non-users (19.3%) was the internet.

## Conclusion

Results of our analyses point to distinct patterns of engagement in health information seeking among CAM users and non-users. CAM users appear to be more active information seekers with relatively greater reliance on traditional information sources than non-users. Our results suggest that CAM users may be a ready audience for patient-centered interventions and cost-effective strategies to engage patients more fully and to improve population health.

